# An Uncommon Case of Pediatric Esthesioneuroblastoma Presenting as SIADH: ^18^F-FDG PET/CT in Staging and Post-Therapeutic Assessment

**DOI:** 10.3390/diagnostics8010008

**Published:** 2018-01-14

**Authors:** Marie Øbro Fosbøl, Anders Bilde, Jeppe Friborg, Eric von Benzon, Andreas Kjær, Christian von Buchwald, Lise Borgwardt

**Affiliations:** 1 Department of Clinical Physiology, Nuclear Medicine & PET, Cluster for Molecular Imaging, Rigshospitalet and University of Copenhagen, DK-2100 Copenhagen, Denmark; eric.von.benzon@regionh.dk (E.v.B.); akjaer@sund.ku.dk (A.K.); lise.borgwardt@regionh.dk (L.B.); 2 Department of ORL, Head & Neck Surgery and Audiology, Rigshospitalet, University of Copenhagen, DK-2100 Copenhagen, Denmark; Anders.Bilde@regionh.dk (A.B.); christian.von.buchwald@regionh.dk (C.v.B.); 3 Department of Clinical Oncology, Rigshospitalet, University of Copenhagen, DK-2100Copenhagen, Denmark; jeppe.friborg@regionh.dk

**Keywords:** esthesioneuroblastoma, ^18^F-FDG-PET/CT, pediatric oncology, paraneoplastic syndromes

## Abstract

Esthesioneuroblastoma (ENB) is an uncommon neuroendocrine tumor originating from the olfactory neuroepithelium and accounts for 3–6% of all intranasal tumors [1]. ENBs can be locally aggressive and cause invasion and destruction of surrounding structures. Histological grading and clinical stage at presentation are highly predictive of survival and especially presence of lymph node and distant metastases are determining prognostic factors [2,3,4,5]. Thus, reliable imaging is essential in these patients. Conventional imaging modalities for staging ENB are magnetic resonance imaging (MRI) and computed tomography (CT). However, fluorine-18 fluoro-2-deoxy-d-glucose positron emission tomography/CT (^18^F-FDG PET/CT) has been reported as a valuable adjunct and was found to upstage 36% of ENB patients compared to conventional imaging [6]. We present a case demonstrating the diagnostic work-up and follow-up with ^18^F-FDG PET/CT in a young patient with ENB with a highly atypical clinical presentation.

**Figure 1:**
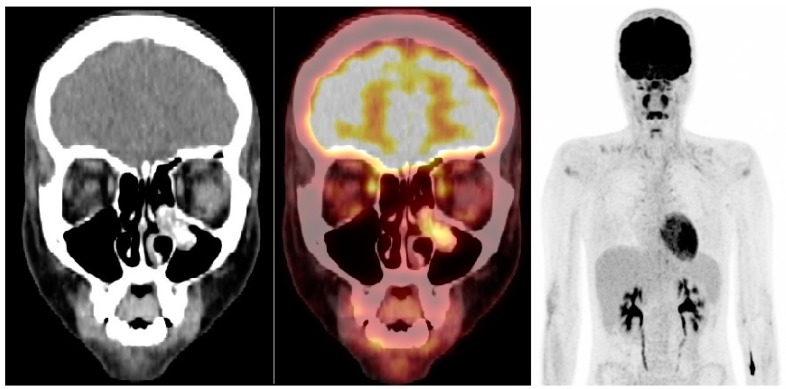
Initial scan—(**left**,**middle**): CT and fused ^18^F-FDG PET/CT, coronal view; (**right**): ^18^F-FDG PET, maximal intensity projection. A 17-year-old girl with no previous medical history was admitted for psychiatric evaluation due to sudden onset of symptoms consisting of hypomania and hallucinations. Prior to admission the patient had suffered from recurrent emesis for a couple of days. As a part of the standard diagnostic work-up, laboratory tests found severe hyponatremia with a serum sodium level of 110 mmol/L (normal range: 135–147 mmol/L), normal serum potassium of 3.7 mmol/L (normal range: 3.3–4.3 mmol/L), low serum ionized calcium 1.14 mmol/L (normal range: 1.18–1.32 mmol/L), high urine sodium level 93 mmol/L (normal range: 20–40 mmol/L) and normal urine osmolarity 406 mmol/kg (normal range 300–900 mmol/kg). The patient was clinically normovolemic and had no abnormal clinical signs besides the psychiatric symptoms. Hypothyroidism and adrenal insufficiency were excluded and syndrome of inappropriate antidiuretic hormone (SIADH) was suspected as the cause of hyponatremia. Treatment with fluid restriction and intravenous isotonic sodium chloride was initialized. In suspicion of an underlying intracranial pathology and as part of standard diagnostic work-up a CT scan of the cerebrum was performed. This revealed no pathological intracranial lesions, but a polypoid mass with calcifications in the left maxillary sinus protruding in to the left nasal cavity. Subsequently, the patient was referred to ^18^F-FDG PET/CT for characterization and staging, which demonstrated the mass in the left maxillary sinus to be highly FDG avid (SUV_max_ 7.4). Besides symmetrical FDG uptake in the pharyngeal lymphatic tissue, which is considered benign, there were no sites of increased FDG uptake in the cervical lymph nodes. Furthermore, no other pathological foci suspicious of metastatic disease were found on the whole-body scan. The tumor was resected en bloc endoscopically under the use of image guidance through a medial maxillectomy. Following surgery, the patient’s symptoms resolved and the electrolytes normalized quickly. Histological and immunohistochemical examination of tumor material were consistent with an ectopic esthesioneuroblastoma Hyams grade 1 positive for chromogranin, synaptophysin, neuron-specific enolase, calretinin, and vasopressin. Histologically the tumor cells reached the margins of the specimen, and the operation was considered non-radical. The patient therefore received post-operative radiation therapy to the surgical area, with no elective neck volumes. Clinical examination and renewed ^18^F-FDG PET/CT scan three months post-treatment showed complete response (Figure 2).

**Figure 2:**
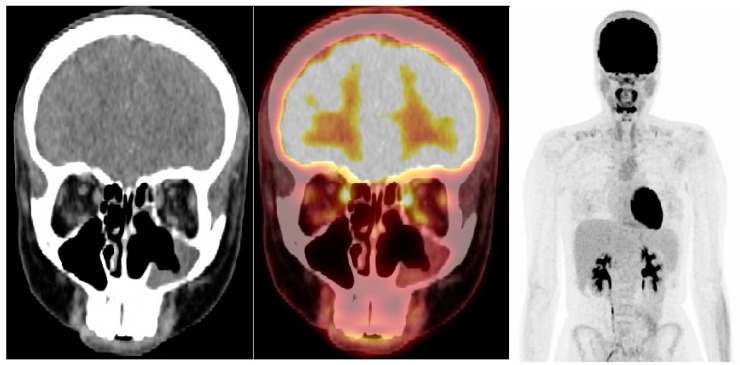
Follow-up scan—(**left**,**middle**): CT and fused ^18^F-FDG PET/CT, coronal view; (**right**): ^18^F-FDG PET, maximal intensity projection. ^18^F-FDG PET/CT 3 months after end of radiation therapy showed no remaining primary tumor or any metastatic disease. There was mildly increased FDG-uptake and mucosal swelling in the upper nasal cavity and frontal sinus, interpreted as an inflammatory reaction to therapy. This case of esthesioneuroblastoma (ENB) is atypical in several aspects. Pediatric cases of ENB are uncommon, as this is a neoplasm most frequently occurring in patients in their 5th and 6th decade [7]. Furthermore, ENBs rarely induce paraneoplastic syndromes and only a few case reports of antidiuretic hormone (ADH) secreting ENBs have been published [8,9,10,11,12,13,14,15,16,17]. Presenting symptoms are usually related to local tumor expansion, such as nasal obstruction and epistaxis [1]. In this case, the disease was diagnosed early due to hyponatremia caused by SIADH, but ENBs are often disseminated at primary staging where cervical lymph node metastases are found in 20–33% of patients. This makes reliable whole-body imaging crucial for these patients. CT is highly valuable in evaluating regional osseous disease, but lacks sensitivity in lymph node metastases and assessment of soft tissue involvement compared to MRI [18]. In a study comparing MRI, ^18^F-FDG PET/CT and endoscopy after primary surgery for ENB in 28 patients, ^18^F-FDG PET/CT was superior to MRI in detecting small lymph node metastases, but did not detect meningeal involvement in three patients which was visualized on MRI [19]. Using ^18^F-FDG PET/MRI in this setting could provide an optimal combination for imaging ENB. Another promising method is somatostatin receptor imaging (SRI) such as ^68^Gallium-DOTATOC PET or ^111^Indium-Octreotide scintigraphy. A subset of ENB expresses somatostatin receptors and the use of SRI in ENB has been described in case reports [20,21,22,23]. Furthermore, radionuclide therapy with somatostatin analogues labeled with beta-emitting isotopes may be a therapeutic option for disseminated ENB [21,22,23]. However, prospective studies are needed to confirm the value of SRI and targeted radionuclide therapy in ENB. In conclusion, ENB is a rare cause of SIADH and other paraneoplastic syndromes, but should be held in mind as the disease may be highly aggressive. Reliable whole-body imaging is important in disease evaluation. As demonstrated in this case, ^18^F-FDG PET/CT can be valuable in staging by excluding or confirming disseminated disease and in follow-up after therapy for ENB.
